# Characterization and formulation into solid dosage forms of a novel bacteriophage lytic against *Klebsiella oxytoca*

**DOI:** 10.1371/journal.pone.0183510

**Published:** 2017-08-17

**Authors:** Teagan L. Brown, Steve Petrovski, Dannielle Hoyle, Hiu Tat Chan, Peter Lock, Joseph Tucci

**Affiliations:** 1 La Trobe Institute for Molecular Science, La Trobe University, Bundoora, VIC, Australia; 2 Department of Physiology, Anatomy and Microbiology, La Trobe University, Bundoora, VIC, Australia; 3 Australian Clinical Labs, Clayton, VIC, Australia; 4 Department of Microbiology, Royal Melbourne Hospital, Parkville, VIC, Australia; Rockefeller University, UNITED STATES

## Abstract

**Aim:**

To isolate and characterize bacteriophage lytic for the opportunistic pathogen *Klebsiella oxytoca* and their formulation into a range of solid dosage forms for *in-vitro* testing.

**Methods and results:**

We report the isolation, genomic and functional characterization of a novel bacteriophage lytic for *Klebsiella oxytoca*, which does not infect the closely related *Klebsiella pneumoniae*. This bacteriophage was formulated into suppositories and troches and shown to be released and lyse underlying *Klebsiella oxytoca* bacteria in an *in-vitro* model. These bacteriophage formulations were stable for at least 49 days at 4°C.

**Conclusions:**

The successful *in-vitro* assay of these formulations here suggests that they could potentially be tested *in-vivo* to determine whether such a therapeutic approach could modulate the gut microbiome, and control *Klebsiella oxytoca* overgrowth, during antibiotic therapy regimes.

**Significance and impact of the study:**

This study reports a novel bacteriophage specific for *Klebsiella oxytoca* which can be formulated into solid dosage forms appropriate for potential delivery in testing as a therapy to modulate gut microbiome during antibiotic therapies.

## Introduction

The genus *Klebsiella* represents a group of organisms which despite contributing to the normal flora, are capable of causing serious disease in man. While people may carry these organisms in their gastrointestinal and upper respiratory tract without any symptoms, these bacteria have the capacity to invade and establish infections in these as well as a range of other tissues, including the lower respiratory tract, urinary tract, liver, central nervous system, circulation, cardiac tissue, dental tissue, and wounds [1–6]. The *Klebsiella* species most commonly associated with human disease are *Klebsiella pneumoniae* and *Klebsiella oxytoca* [7]. More recently there have been reports to suggest that *K*. *oxytoca* is the causative agent in a significant proportion (up to 50%) of antibiotic associated haemorrhagic colitis [8, 9], and antibiotic associated diarrhoea and may be underdiagnosed because of the lack of sensitivity of the routine laboratory culture media [10]. The *K*. *oxytoca* associated colitis and diarrhoea therefore can significantly decrease the quality of life of the patient and their capacity to tolerated antibiotic regimens [8, 9].

A factor that may contribute to the haemorrhagic colitis and diarrhoea caused by *K*. *oxytoca* following antibiotic treatment is that this bacterium is known to carry genes for resistance to an array of antibiotics [3, 4]. Its armoury includes chromosomal genes for OXY beta-lactamases and efflux mechanisms such as AcrAb [11] which may confer resistance against later generation cephalosporins [4, 12]; and plasmid mediated resistance genes, such as metallo-beta-lactamases, which may enable resistance against carbapenems [13, 14]. The environment created under the selective pressure of antibiotic regimes may allow for the specific survival and overgrowth of *K*. *oxytoca*, which is known to constitutively express some of these drug resistance mechanisms [9].

To alleviate symptoms of antibiotic induced colitis and diarrhoea, modulation of intestinal flora represents a potentially useful therapeutic target. While previously demonstrated in animals, very recent studies have shown that faecal microbiota transplantation in humans could minimise the gut colonisation by antibiotic resistant organisms following antibiotic therapy [13, 15]. Other less invasive approaches include the use of probiotics, but whilst these have been demonstrated to be efficacious and safe in minimising diarrhoea during antibiotic therapy [16, 17], they are not without their adverse reactions [17]. The current use of probiotic modulators of intestinal flora is extensive, with the value of the world market estimated at approximately $35 billion US [18]. As these products are not covered by Pharmaceutical Benefit Schemes, however, the financial cost to the individual patient when using high dosage probiotics is significant.

A possible alternative to the approaches described above for the treatment of antibiotic associated colitis and diarrhoea is the use of lytic bacteriophage (phage), viruses which are endogenous killers of bacterial cells [19, 20]. The application of phages to treat bacterial infections is an attractive prospect. Firstly, their action is very specific, as they lyse only their bacterial hosts. Further, they display single-hit kinetics and are auto “dosing” in that their replication at the site of infection leads to marked increases in their titre [21]. Importantly, phages are generally regarded as medically and environmentally safe, bring about minimal disruptions to the autogenous microbial community, and will lyse antibiotic resistant strains [21]. These last two features are particularly relevant in the potential treatment of *K*. *oxytoca*. In 2014, the National Institutes of Health in the U.S. suggested that use of phage was among one of the more innovative and promising components of strategies to combat antimicrobial resistance [22].

To date, a diverse range of applications for phage therapy have been reported, with dosage forms ranging from injections (cutaneous, intravenous, subcutaneous, intrapleural) to liquids [23–25]. We and others have reported the potential of semi-solid preparations for the delivery of phages [26–28]. More recently we tested the efficacy, stability and optimal storage conditions of a range of semi-solid and solid dosage forms for the delivery of lytic phages in an in-vitro model [29]. While several phages that are capable of lysing *K*. *oxytoca* have been reported, these have been propagated on *K*. *pneumoniae* and suggested to have a host range that extends to *K*. *oxytoca* [30, 31]. Here we report the isolation, as well as genomic and functional characterization, of novel and unique phage that was propagated on *K*. *oxytoca*, and the subsequent formulation of these into solid dosage forms, namely a troche (or lozenge), and a suppository. These *K*. *oxytoca* phage formulations were then assayed and shown to be capable of releasing the phage so that there was lysis of the underlying *K*. *oxytoca* bacteria in an in-vitro model. The potential benefit of such formulations in the treatment of antibiotic induced *K*. *oxytoca* mediated colitis and diarrhoea is discussed.

## Material and methods

### Bacterial cultures and identification

*Klebsiella* cultures had been previously isolated from human subjects for other purposes.

All samples were obtained with oral consent, de-identified and handled according to the La Trobe University Ethics committee. No ethical concerns were raised as the human material was not the focus of the study and bacteria obtained were not traced back to the individual.

All cultures were grown using Tryptic Soya broth and agar (Oxoid, Adelaide, Australia). The cultures were incubated at 37°C under anaerobic conditions using the Anaerogen system (Oxoid). Matrix assisted laser desorption ionisation- time of flight (MALDI-TOF) technology was used to initially identify cultures. Isolated single colonies were subjected to biochemical testing and confirmation of identification using the Microbact 24E system (Oxoid), according to the manufacturers’ instructions. For unequivocal identification, PCR amplification of the 16S rRNA gene of each bacterial strain was performed using universal primers 27F, 519R, 895F and 1492R [32–34]. PCR products were cleaned using Ultra Clean® DNA purification kit (MO-BIO, California, USA), and the DNA analysed by Sanger sequencing at the Australian Genome Research Facility (Brisbane, Australia). A feature unique to *K*. *oxytoca* strains, which is not seen in *K*. *pneumoniae*, is the capacity to degrade pectin (via excretion of polygalacturonases), and so as a final differentiation between these species, PCR was performed to test for the presence of the polygalacturonase (pehX) gene [35].

### Isolation and purification of phage

A lawn of *K*. *oxytoca* was prepared using a cotton swab, then a 10 μL sample of wastewater (collected from the Bendigo Wastewater Treatment Plant; Bendigo, Australia) was placed directly onto the lawn surface and the plate incubated for 2 days. If clearing was observed, the plaque was excised along with the agar, resuspended in 500 μL of broth and centrifuged (17,000 x *g* for 5 min) as described previously [36]. The supernatant was removed, placed in a fresh tube and a 10-fold serial dilution of the phage performed. Each dilution was spotted directly onto a lawn of the sensitive *K*. *oxytoca* bacteria and plaques were observed. This purification process was repeated five times to ensure that each plaque resulted from a single infecting virion.

#### Extraction of phage DNA

As described in detail previously [26], five mL of phage lysate (>10^9^ PFU mL^-1^) was treated with MgCl_2_ (final concentration 5 mM), DNase I and RNase A (final concentrations of 10 μg mL^-1^) for 30 minutes at room temperature. Phage virions were recovered by polyethylene glycol (PEG) precipitation using PEG 8000 and sodium chloride (final concentrations 10% wt/vol, and 1 M respectively) at 4°C overnight. The precipitate was centrifuged (17,000 × ***g*** for 15 min) and the pellet resuspended in nuclease free water (Promega, Sydney, Australia). Phage were exposed to Proteinase K (final concentration 50 μg mL^-1^), EDTA (final concentration 20 mM) and sodium dodecyl sulphate (final concentration of 0.5% [vol/vol]) at 55°C for 1 hour. An equal volume of phenol-chloroform-isoamyl alcohol (29:28:1) was added and thoroughly mixed before being centrifuged at 12,000 × ***g*** for three minutes. The aqueous phase was carefully removed and an equal volume of isopropanol and 1/10 volume sodium acetate (pH 5) was added. The mixture was left at -20°C overnight to allow DNA precipitation. After centrifugation (12,000 × ***g*** for 10 min) the supernatant was removed, the DNA pellet was washed with 70% ethanol, air dried and resuspended in 30 μl of nuclease free water (Promega, Sydney, Australia).

#### Phage sequencing and annotation

As described in detail previously [26], extracted phage DNA was prepared for sequencing using a Nextera® XT DNA sample preparation kit as per the manufacturer’s instructions. The DNA libraries were sequenced using a Miseq® V2 reagent kit (300 cycles) on an Illumina MiSeq® as 150 bp paired end reads. Sequenced reads were assembled *de novo* using CLC workbench (Qiagen, Version 8.5.1), and open reading frames predicted using Glimmer (version 3.02). ARAGORN [37] and tRNAscan-SE [38] were used to predict tRNA and tmRNA present in the sequences. The full genomic nucleotide sequence of the *K*. *oxytoca* phage was deposited into GenBank under accession number KY780482.

### Transmission electron microscopy

Phage particles were allowed to adsorb to 400-mesh formvar and carbon coated copper grids (ProSciTech, Townsville, Australia) for 5 minutes. Excess solution was removed using filter paper before the grids were negatively stained with three 20 sec applications of 2% [wt/vol] uranyl acetate (Sigma, Sydney, Australia) with removal of excess stain on filter paper after each application. Grids were air-dried for 20 minutes before examination under a JEOL JEM-2100 transmission electron microscope (TEM) operated at an accelerating voltage of 200 kV. High resolution digital images were recorded on a Gatan Orius SC200D 1 wide angle camera with Gatan Microscopy Suite and Digital Micrograph (Version 2.32.888.0) imaging software. Viruses were measured using ImageJ software.

### *K*. *oxytoca* phage host range

To assess whether the phage could lyse other *Klebsiella* strains, a serial dilution series of the phage stock (~10^9^ PFU mL^-1^) was completed. 10 μL of each dilution was spotted directly onto a lawn culture of the *Klebsiella* strains which had been identified as described above (Table 1). *Klebsiella oxytoca* (ATCC 13182) and *Klebsiella pneumoniae* (ATCC 13883) obtained from the American Type Culture Collection (Washington DC, USA) were also used in the host range experiments, as were *Pseudomonas aeruginosa* and *Escherichia coli* (which were part of our laboratory culture collection). The observation of plaques on the lawn culture indicated that the phage was able to infect and lyse the host. The PFU (counts per mL) for each strain were also compared to that of the original host to determine the relative efficiency of plating.

**Table 1:** Identification of the isolated *Klebsiella* species.

Lab code	MALDI-TOF Identification	Microbact Identification^a^	16S sequencing (closest match)^b^	PehX gene
KLEB001	*K*. *pneumoniae*	*K*. *pneumoniae* Code: 47563776	*K*. *pneumoniae* strain DSM 30104	Negative
KLEB002	*K*. *pneumoniae*	*K*. *pneumoniae* Code: 47562766	*K*. *pneumoniae* strain DSM 30104	Negative
KLEB003	*K*. *oxytoca*	*K*. *oxytoca* Code: 47763766	*K*. *oxytoca* ATCC 13182	Positive
KLEB004	*K*. *oxytoca*	*K*. *oxytoca* Code: 47763776	*K*. *oxytoca* ATCC 13182	Positive
KLEB005	*K*. *oxytoca*	*K*. *oxytoca* Code: 47761376	*K*. *oxytoca* ATCC 13182	Positive
KLEB006	*K*. *pneumoniae*	*K*. *pneumoniae* Code: 47563766	*K*. *pneumoniae* strain DSM 30104	Negative
KLEB007	*K*. *pneumoniae*	*K*. *pneumoniae* Code:47563776	*K*. *pneumoniae* strain DSM 30104	Negative
KLEB008	*K*. *oxytoca*	*K*. *oxytoca* Code: 47763766	*K*. *oxytoca* ATCC 13182	Positive
KLEB009	*K*. *pneumoniae*	*K*. *pneumoniae* Code: 47563776	*K*. *pneumoniae* strain DSM 30104	Negative
KLEB010	*K*. *oxytoca*	*K*. *oxytoca* Code: 47763766	*K*. *oxytoca* ATCC 13182	Positive

^a^ Microbact database search using the octal code.

^b^NCBI Blast analysis using the 16S rRNA sequences (Bacteria and Archaea).

### One step growth curve

As described in detail previously [26], a phage stock of ~10^9^ PFU mL^-1^ was added to an exponential phase broth culture of *K*. *oxytoca* (propagation host), to a multiplicity of infection (MOI) of 0.1. After a 10 minute adsorption period [31] the cells were washed twice with broth and resuspended in 1 mL of fresh broth. To minimise the amount of non-adsorbed phage this culture was then diluted 1 in 100. 100 μL samples were taken every 10 minutes to calculate viable phage concentrations (PFU mL^-1^) [31, 39].

### *K*. *oxytoca* phage stability in simulated gastric fluid and bile salts solution

To test phage stability in simulated gastric conditions, a phage stock at a concentration of ~10^9^ PFU mL^-1^ in simulated gastric fluid [40] with 0.32% (wt/vol) pepsin at pH 2.5 was used. 100 μL samples were taken every 15 minutes to calculate viable phage concentrations (PFU mL^-1^). The same experiment was carried out with 0.5% bile salts (OXOID) in intestinal test solution [40] at pH 6.8 to determine the viability of phage when exposed to emulsifying bile salts.

### *K*. *oxytoca* phage formulation into suppositories

Suppositories were manufactured according to established formularies [41] with a PBS phage stock at a concentration greater than 10^9^ PFU mL^-1^. The suppositories were formulated as follows: gelatin powder and purified water were heated at 100°C until dissolved, before glycerol was added according to the formulation. The product was then cooled to 40 degrees Celsius and the phage was added to a final concentration of 4.5x10^8^ PFU per gram of formulation. The formulation was deposited into 1.2 mL silicon molds. To test if the phage were capable of lysing their host bacteria, the suppository was sliced along its longitudinal axis to generate two equal halves, and these were then placed flat side down on an agar lawn of *K*. *oxytoca* bacteria. The plates were incubated at 37°C for 2 days and any lysis of the bacteria in the presence of the suppository was indicated by a clear zone.

### *K*. *oxytoca* phage formulation into troches

Troches were manufactured according to a formula from the Professional Compounding Chemists of Australia. Silca gel (micronized), stevioside powder, acacia powder and citric acid anhydrous powder were triturated and sieved, before being added to melted polyethylene glycol base (PEG base, PCCA). The formulation was mixed until homogenous, cooled and the phage was added to a final concentration of 4.5x10^8^ PFU per gram of formulation. The product was poured into troche molds and allowed to set. The troches were then tested by placing the troche flat onto a lawn of *K*. *oxytoca* bacteria. The plates were incubated at 37°C for 2 days and any lysis of the bacteria in the presence of the troche was indicated by a clear zone.

### Stability of the *K*. *oxytoca* phage suppositories and troches

Following their formulation, the phage suppositories and troches were stored at 4°C, protected from light. A qualitative assessment of the capacity for phages to kill *K*. *oxytoca* bacteria was conducted at weekly intervals as detailed above. For the pessary, a quantitative assessment was also conducted. The phage pessary was solubilised in preheated PBS (37°C) at a dilution of 1 in 10 and then used to assess the concentration of viable phage (PFU mL^-1^). Such quantitation was not possible with the phage troche, as there was precipitation of material during the solubilisation process.

## Results

### Identification of *Klebsiella* strains

Five *K*. *oxytoca* and five *K*. *pneumoniae* strains were identified as detailed in Table 1. Biochemical analysis (Microbact 24E) revealed that all strains could use glucose, mannitol, xylose, citrate, inositol, rhamnose, sucrose, lactose, arabinose, raffinose and salicin as the sole carbon source. All strains were positive for lysine decarboxylase, acetoin production (VP reaction), ONPG and urease hydrolysis. *K*. *oxytoca* strains were indole positive whereas *K*. *pneumoniae* strains were negative. PCR amplification of the pehX gene was observed in only the *K*. *oxytoca* stains (Table 1).

### Isolation of the phage and its genome

**Fig 1:**
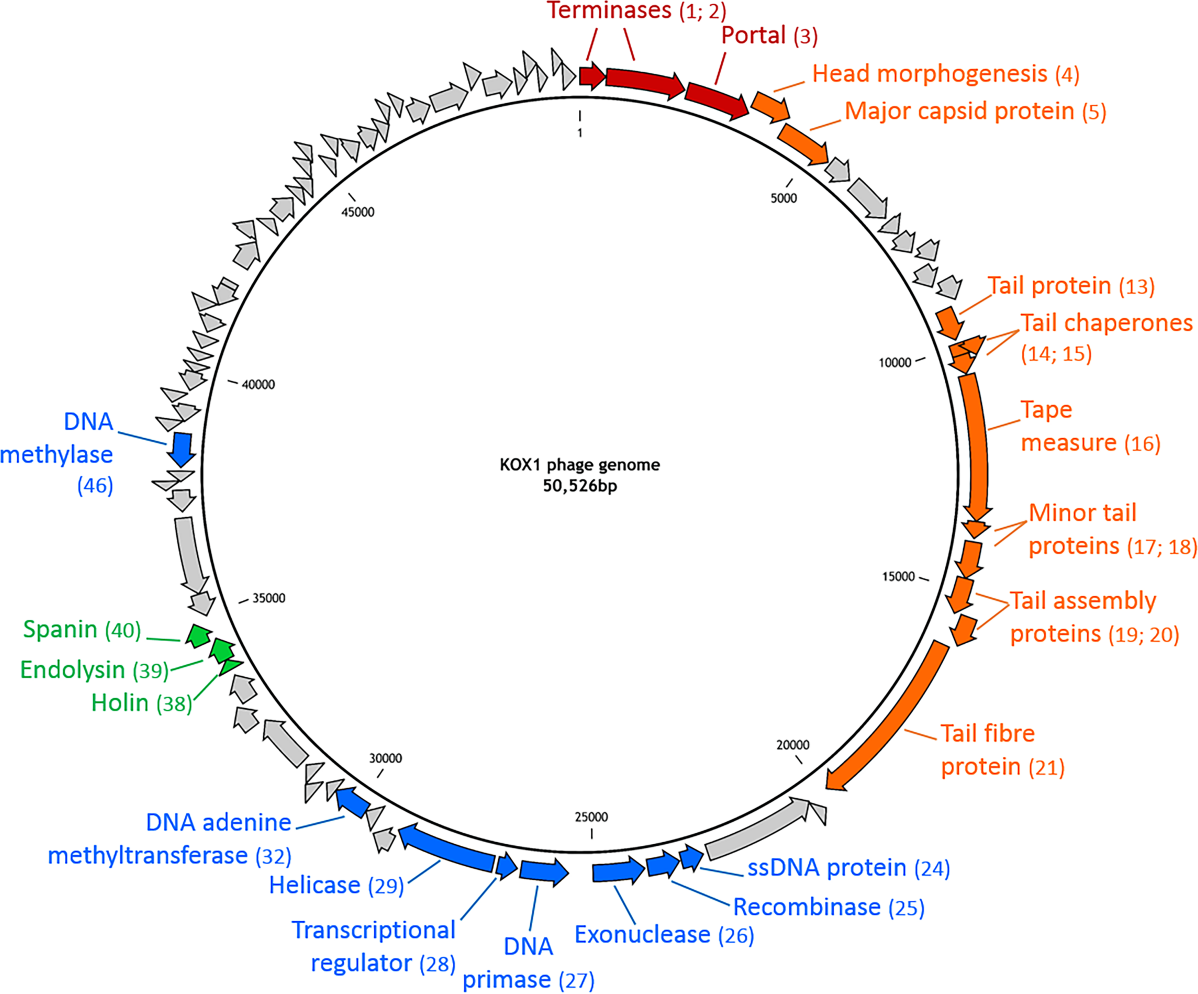
KOX1 genome. The arrows indicate the ORFs predicted from the sequence and the direction of their translation. The putative function and the ORF number is noted. Red arrows indicate the putative packaging genes, orange arrows are putative structural genes for the capsid and tail morphology, blue arrows are putative DNA manipulation genes and the green arrows indicate putative lysis genes. Grey arrows are ORFs with no ascribed function.

Following screening with samples from wastewater treatment plants, visible lytic plaques were detected on KLEB010, one of the *K*. *oxytoca* strains identified in this study. These plaques were less than one millimetre in diameter with no visible ‘halo’ or other disturbance to the surrounding bacterial lawn. The plaques were purified and a stock generated by propagating on KLEB010. DNA was extracted and sequenced (with coverage of over 600 times) using the Illumina MiSeq next generation sequencing platform and the reads were assembled *de novo* with CLC Genomics workbench programs. This *K*. *oxytoca* phage, termed KOX1, had a genome of 50,526 base pairs in length (Fig 1). KOX1 had a circular dsDNA genome with an average GC content of 51.2% which is lower than that of its host *K*. *oxytoca* (~55%). There were 81 open reading frames (ORFs) predicted from the sequence (see Fig 1). Analysis of the ORFs revealed that only 30% (25 out of 81) of these could be assigned a putative function (see Table 2). For our presentation, ORFs were labelled from the putative terminases and the genes appeared in a modular structure (Fig 1). All of the ORFs with a putative ascribed function, including terminases, portal proteins, tail structural proteins, lysis proteins, recombinases and DNA manipulation genes showed close homology to previously isolated *Klebsiella* phage (Table 2). There were no tRNA or tmRNA detected in the sequence.

**Table 2:** Summary of KOX1 ORFs with predicted protein function.

ORF	Polypeptide length	Predicted function (conserved motif)^a^	Closest match^b^	E-value^c^
1	175	Putative terminase small subunit (pfam16677)	Klebsiella phage PKP126	3e-126
2	535	Putative terminase large subunit (pfam03237)	Klebsiella phage PKP126	0
3	440	Putative portal protein	Klebsiella phage vB_KpnS_KpV522	0
4	257	Putative head morphogenesis protein (pfam04233)	Klebsiella phage vB_KpnS_KpV522	0
5	377	Putative major capsid protein	Klebsiella phage PKP126	0
13	218	Putative major tail protein (pfam08813)	Klebsiella phage PKP126	2e-155
14	109	Putative tail assembly chaperone (pfam08748)	Klebsiella phage PKP126	3e-76
15	214	Putative tape measure chaperone (pfam08748)	Klebsiella phage KLPN1	4e-125
16	993	Putative tape measure protein (pfam06791 and pfam09718)	Klebsiella phage PKP126	0
17	115	Putative minor tail protein (pfam05939)	Klebsiella phage PKP126	5e-78
18	251	Putative minor tail protein (pfam05100)	Klebsiella phage PKP126	0
19	246	Putative tail assembly protein	Klebsiella phage vB_KpnS_KpV522	0
20	201	Putative tail assembly protein (pfam06805)	Klebsiella phage KLPN1	3e-141
21	1257	Putative tail fibre protein	Klebsiella phage KLPN1	0
24	154	Putative ssDNA binding protein	Klebsiella phage PKP126	7e-96
25	219	Putative recombination protein (pfam04404)	Klebsiella phage PKP126	2e-161
26	349	Putative exonuclase (pfam12684)	Klebsiella phage vB_KpnS_KpV522	0
27	321	Putative DNA primase (pfam08273)	Klebsiella phage PKP126	0
28	134	Putative transcriptional regulator (pfam14549)	Klebsiella phage PKP126	1e-93
29	679	Putative DNA helicase (pfam00271)	Klebsiella phage vB_KpnS_KpV522	0
32	244	Putative DNA adenine methyltransferase (pfam05869)	Klebsiella phage PKP126	4e-178
38	72	Putative holin	Klebsiella phage vB_KpnS_KpV522	3e-41
39	161	Putative endolysin (pfam00959)	Klebsiella phage vB_KpnS_KpV522	2e-115
40	142	Putative spanin	Klebsiella phage vB_KpnS_KpV522	2e-110
46	231	Putative DNA methylase	Klebsiella phage vB_KpnS_KpV522	6e-170

*a* Predicted function is based on amino acid identity, conserved motifs, N-terminal sequencing, and gene location within functional modules.

*b* The most closely related gene and the name of the organism.

*c* The probability of obtaining a match by chance as determined by BLAST analysis. Only values of less than 10^−4^ were considered significant

Note: The ORFs not listed in this table code for proteins with unknown function as determined by BLAST analysis.

### KOX1 characterization

**Fig 2:**
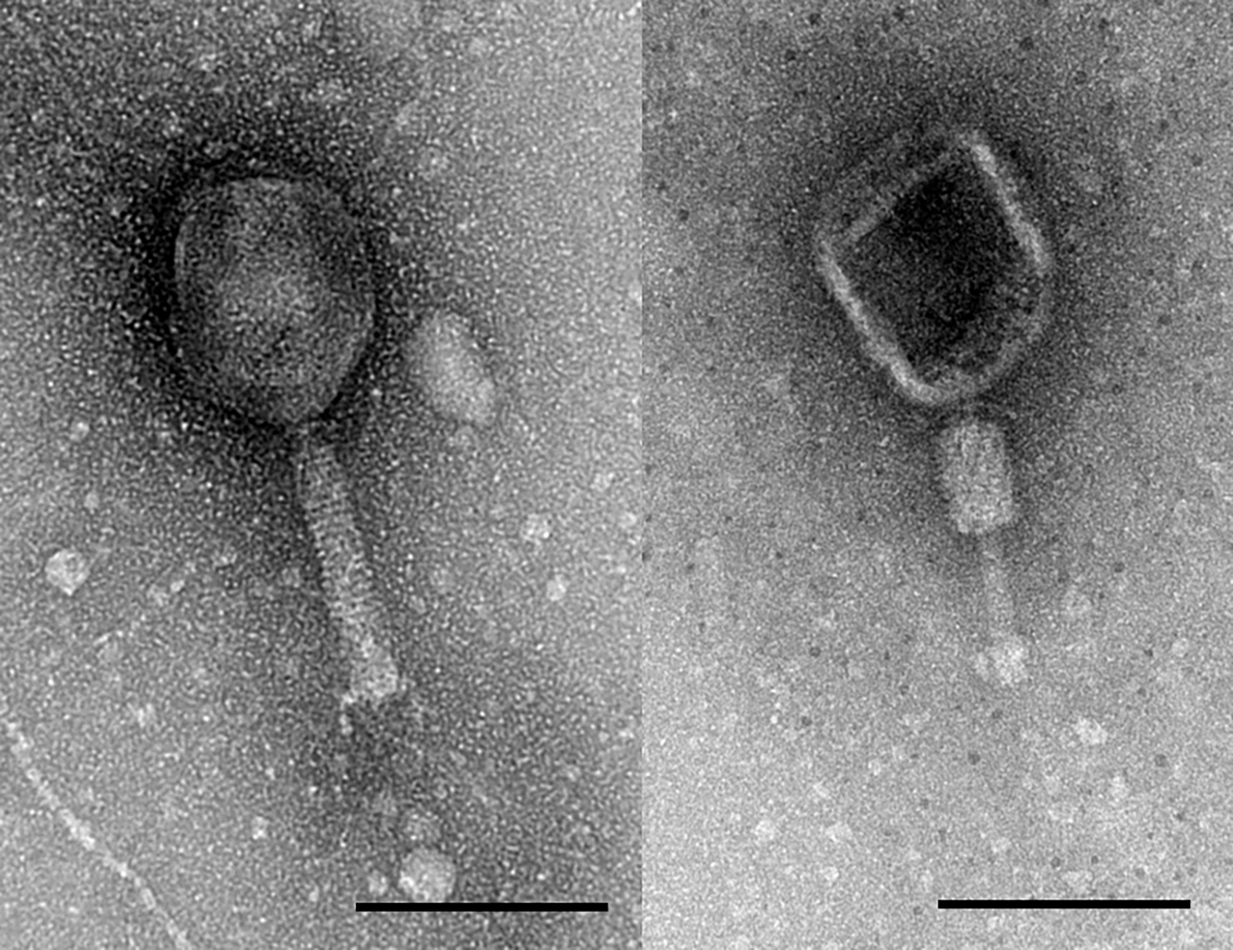
Transmission electron microscopy of KOX1. The image on the right shows the phage tail contracted. The scale bars represent 100 nm.

**Fig 3:**
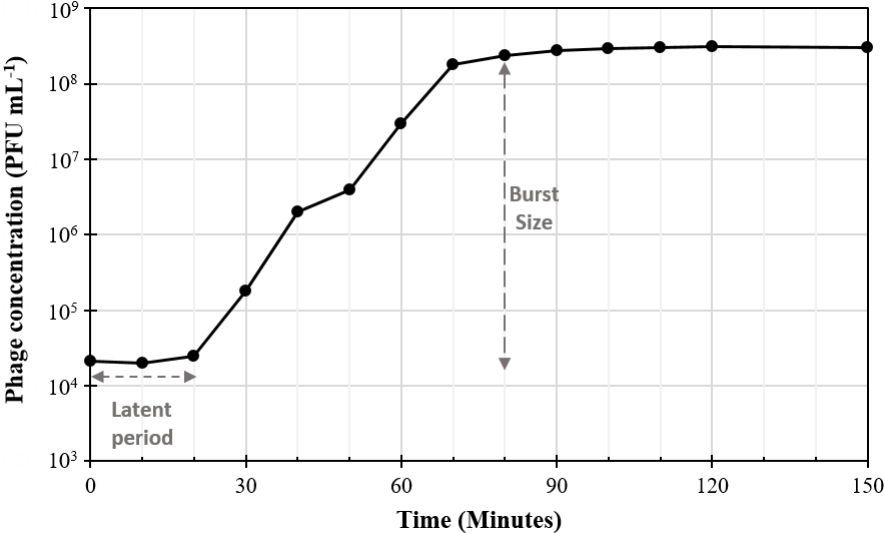
One step growth curve for KOX1. Depicted is the population burst size and the latent period (See S1 Table for values).

Transmission electron microscopy of KOX1 revealed it belonged to the *Myoviridae* family with a prolate icosahedral head (110 ±2 nm by 80 ±2 nm) and contractile tail (115 ±2 nm) placing the phage in the order *Caudovirales* (Fig 2). KOX1 was found to be lytic only on *K*. *oxytoca* strains and didn’t lyse *K*. *pneumoniae*, *Escherichia coli* or *Pseudomonas aeruginosa* strains. The phage was able to lyse five of the six *K*. *oxytoca* strains isolated in this study, these being KLEB003, KLEB004, KLEB008 and KLEB010, as well as the ATCC obtained *K*. *oxytoca*. The plaquing efficiency on KLEB008 and ATCC 13182 *K*. *oxytoca* was equal to that of the original host (KLEB010; ~10^8^ PFU mL^-1^), whereas on KLEB003 and KLEB004 the plaquing efficiency was an order of magnitude less (~10^7^ PFU mL^-1^). While inhibition of growth on the *K*. *pneumoniae* strains (ATCC 13883 and KLEB001) was observed at high phage titres, plaques failed to be produced. One step growth experiments showed that KOX1 had a latent period of 20 minutes and the population burst was 3.0x10^8^ PFU mL^-1^, which equated to a burst size of 35 PFU per infected bacteria (Fig 3; S1 Table). Our observations of the latent period concur with those previously demonstrated on *K*. *oxytoca* [30, 31]. However, the previously reported burst sizes (measured in PFU per infected cell) were lower than what we estimate here [30].

### Phage stability in simulated gastric fluid and bile salts solution

**Fig 4:**
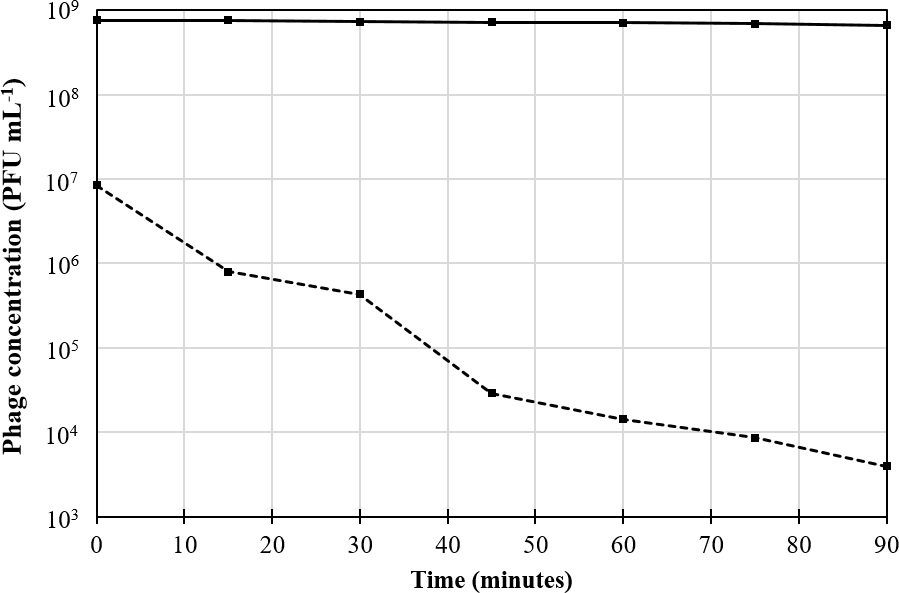
KOX1 stability in simulated gastric fluid and bile salts solution. The stability over time for each is indicated by the broken and solid lines respectively. The y-axis is in log scale, with data points representing the mean of three samples. Because the measurements for each of the triplicates were very similar, it was not possible to adequately represent the error bars for standard deviation in the figure (see S2 Table for values).

As depicted in Fig 4, even after 90 minutes exposure, phage viability was minimally affected by bile salts. Upon immediate exposure to simulated gastric fluid (pH 2.5), phage viability was reduced by two orders of magnitude. Under these conditions, there was a steady decrease in phage numbers, until after 90 minutes exposure, viable phage numbers had dropped to under 10^4^ PFU mL^-1^ (Fig 4; S2 Table).

### KOX1 formulation into solid dosage forms and capacity to lyse *K*. *oxytoca in vitro*

**Fig 5:**
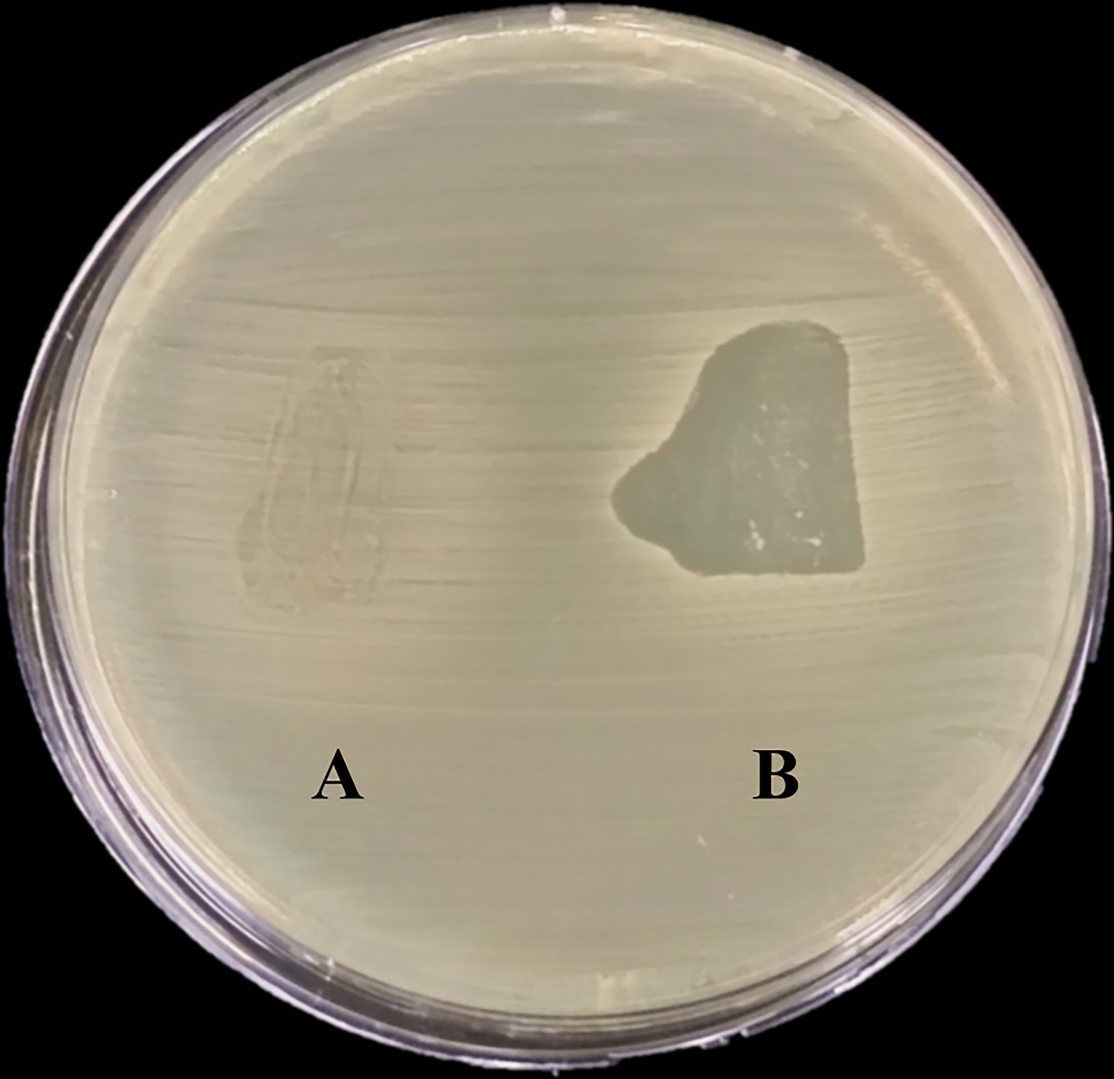
KOX1 phage lytic activity in a suppository. The left side of the plate (A) shows effect on growth when a control suppository, with no phage formulated is placed on the bacterial lawn. The right side of the plate (B) shows effect on bacterial growth when a suppository formulated with phage at a final concentration of 4.5x10^8^ PFU per gram of formulation is placed on the lawn. While there was some effect seen in (A), most likely due to bacterial disturbance when placing the suppository, the phage lytic activity is clearly observed in B.

**Fig 6:**
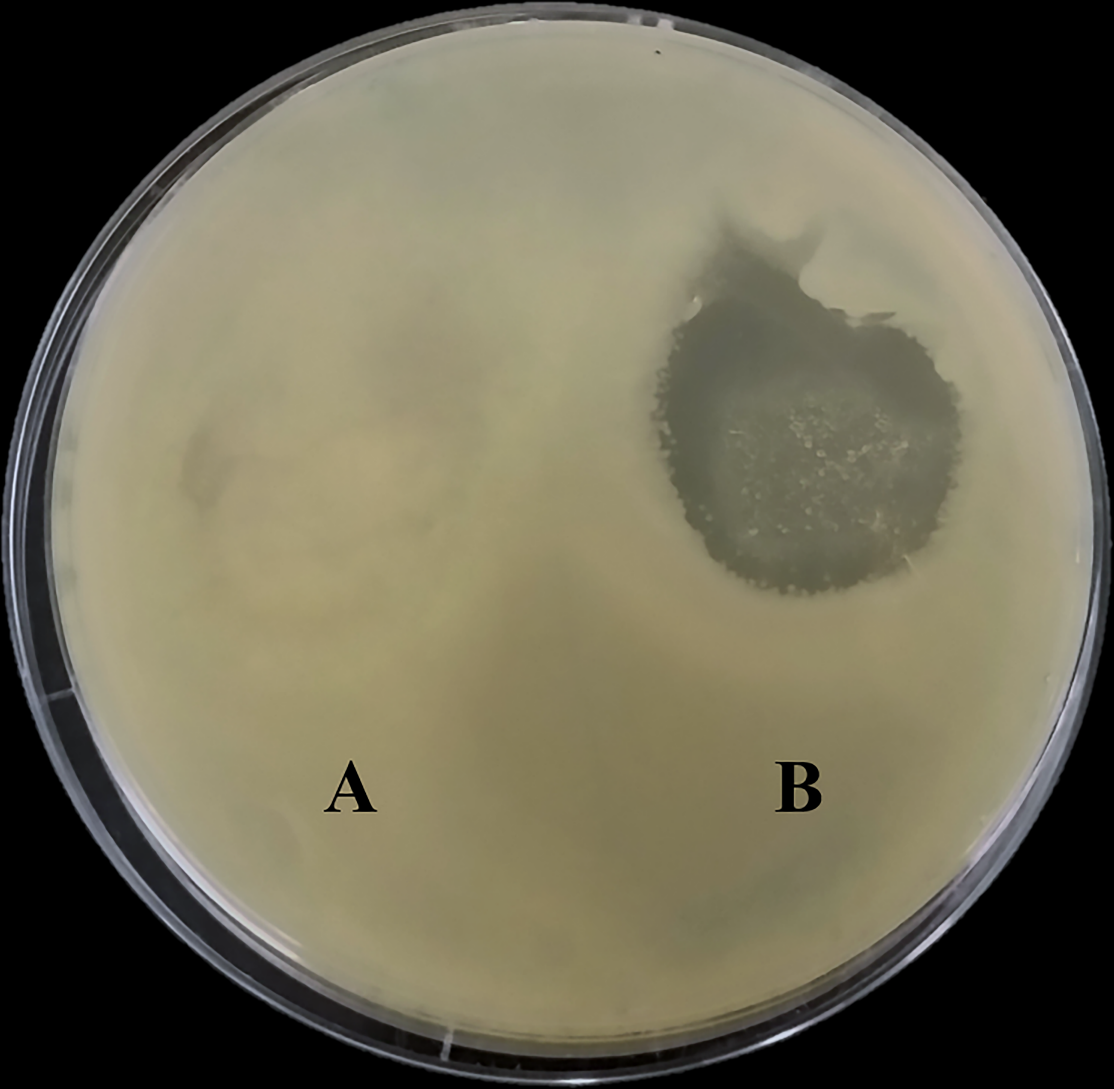
KOX1 phage lytic activity in a troche. The left side of the plate (A) shows effect on growth when a control troche, with no phage formulated is placed on the bacterial lawn. The right side of the plate (B) shows effect on bacterial growth when a troche formulated with phage at a final concentration of 4.5x10^8^ PFU per gram of formulation is placed on the lawn. While there was some effect seen in (A), most likely due to bacterial disturbance when placing the troche, the phage lytic activity is clearly observed in B.

**Fig 7:**
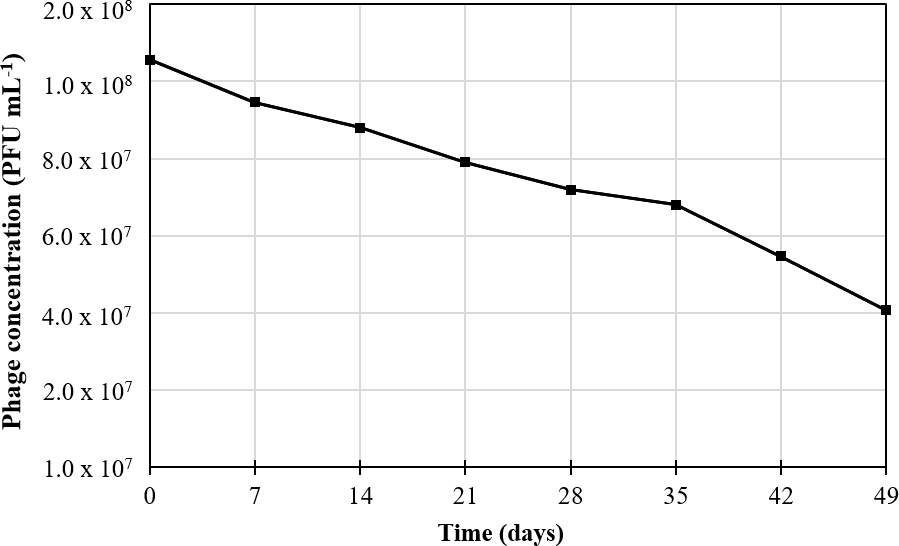
Quantitation of KOX1 phage suppository lytic activity (see S3 Table for values).

KOX1 phage were formulated into suppositories and troches, which were placed upon lawns of *K*. *oxytoca* bacteria on culture plates. Both formulations were capable of releasing the phage to lyse the underlying bacteria (Fig 5 and Fig 6). These formulations were tested weekly, and when stored at 4°C the phage suppositories maintained lytic activity for at least 49 days (Fig 7) and the phage troche maintained lytic activity for 56 days (as visualised by clearing in-vitro). Fig 7 shows that 49 days after formulation, suppositories contained viable phage at a concentration of greater than 10^7^ PFU mL^-1^ (S3 Table).

## Discussion

This study aimed to isolate and characterize phage which were lytic against *Klebsiella oxytoca*, and formulate them into dosage forms which could potentially be tested in the control of the gut microbiome during antibiotic therapy. While differentiation between the *Klebsiella* species *pneumoniae* and *oxytoca* is not always straightforward [35, 42], in the current study we employed data from the complete sequence of the 16S gene, Microbact® biochemical testing, MALDI-TOF analysis and PCR amplification of the pehX gene from isolated strains in order to obtain a definitive identification. The phage which have been reported to target *Klebsiella* to date show a diverse range of morphology including *Podoviradae* (51% of isolates), *Myoviridae* (33% of isolates) and *Siphoviridae* (16% of isolates) (NCBI nucleotide database, https://www.ncbi.nlm.nih.gov/nucleotide/). Of those which are reported as targeting *K*. *oxytoca*, most belong to the Myoviridae family, with genomes ranging in size from 168 to 174 Kb [30, 31, 43]. KOX1, the unique *K*. *oxytoca* lytic phage isolated here, belongs to the *Myoviridae* family, but has a much smaller genome of approximately 50.5 Kb. The uniqueness of this phage is underlined by the fact that less than 30% of its ORFs have similarity to genes with any known functionality.

While it has been reported that phage isolated on and lytic against *K*. *pneumoniae* were able to lyse strains of *K*. *oxytoca* [30, 31, 43], KOX1 was specific for *K*. *oxytoca* bacteria, and did not readily lyse the *K*. *pneumoniae* strains tested here. Yet KOX1 was not without some activity against the *K*. *pneumoniae* strains ATCC 13883 and KLEB001. Here, while there was some bacterial lysis at high phage titres, plaques failed to be produced. *K*. *pneumoniae* strains which are closely related to those isolated and used here, (such as that depicted by Accession number CP014294) do not appear to contain the conserved BREX gene cassette, nor elements of the Argonaute pathway [44], both of which confer resistance to a range of phage. One possibility is that the KOX1 phage bound to the *K*. *pneumoniae* strain and initiated lysis, but then the phenomenon of abortive infection subsequently restricted the production of phage progeny to the extent that plaques were not observed. Additionally, there is some evidence of CRISPR/Cas adaptive immunity systems present in some *K*. *pneumoniae* strains which potentially limit phage infection [45].

We have shown here that the KOX1 phage was able to be formulated into solid dosage forms such as troches and suppositories, and that these formulations were stably maintained for a period of up to 49 and 56 days, respectively, at 4°C. These phage formulations displayed *in-vitro* lytic capacity against *K*. *oxytoca* for up to this period. Quantitative analysis of the phage suppository showed that greater than 10^7^ viable phage could be isolated following 49 days storage. Such formulations could potentially be tested for use in the treatment of antibiotic associated haemorrhagic colitis and diarrhoea caused by *K*. *oxytoca*. While these gut pathologies are reversible and symptoms subside following cessation of antibiotic therapy [4, 6, 12], there is significant impact on the patient quality of life during this period [4, 12], and this is potentially exacerbated with antibiotic regimes that continue for extended periods of time. Further, the cost to the patient of products to modulate the intestinal flora during antibiotic therapy, such as probiotics, is significant, while procedures such as faecal microbiota transplantation are invasive and as yet in their infancy. The ingestion of troches or administration of suppositories with *K*. *oxytoca* lytic phages as the medicament could prove a useful way to modulate intestinal flora, and assist in reducing overgrowth of antibiotic resistant *K*. *oxytoca* strains, during antibiotic therapy. To this end, we have shown that the KOX1 phage is capable of surviving in environments simulating the gastric chamber. Specifically, our experiments showed that following 30 minutes exposure to these conditions, in which time the phage may be expected to have passed through the stomach if ingested via a troche, there are still significant numbers (10^5–^10^6^ PFU mL^-1^) of viable phage. As low gastric pH is a significant contributor to the reduction in phage numbers, the co-administration of proton pump inhibitor therapy, such as omeprazole, may be used in order to enhance phage viability. Such medication is commonly used for gastric reflux, and has the capacity to decrease stomach acidity to pH 6 [46]. This approach may negate the need for microencapsulation of phage into microspheres, which has been suggested as a means of formulating phage to improve viability following traffic through the gastric chamber [47]. Once viable phage have passaged through the stomach, they will encounter bile salts in the duodenum, but as we have shown here, they are not likely to be adversely affected by such emulsifying agents. If the oral route of delivery for the *K*. *oxytoca* phage, via troches, proves difficult, then administering the phage in a suppository, as we have formulated here, may be preferable.

## Conclusion

We report here the isolation and characterization of a unique lytic phage for *K*. *oxytoca*, which is capable of being stably formulated into solid dosage forms. The *in-vitro* lytic capacity of these formulations against *K*. *oxytoca* bacteria is maintained for at least 49 days, when stored at 4°C. This study may provide the basis for clinical testing of these *K*. *oxytoca* phage formulations in order to modulate the intestinal microbiota during antibiotic therapy, and thus reduce the GI effects associated with *K*. *oxytoca* overgrowth.

## Supporting information

S1 Table The values shown in S1 Table were used to generate Fig 3. This experiment was completed on three additional occasions with similar results.
(DOCX)

S2 Table Because the measurements for each of the triplicates in Fig 4 were very similar, it was not possible to adequately represent the error bars for standard deviation. The values shown in S2 Table were calculated and used to generate Fig 4.
(DOCX)

S3 Table These values were used to generate Fig 7. (DOCX)
