# Supplementary material for: Characterization and formulation into solid dosage forms of a novel bacteriophage lytic against Klebsiella oxytoca
Source: PLoS One. 2017 Aug 17;12(8):e0183510. doi: 10.1371/journal.pone.0183510 (PMC5560551; doi:10.1371/journal.pone.0183510)
Supplement: S1 Table — This experiment was completed on three additional occasions with similar results. (DOCX) [file pone.0183510.s001.docx]

**Supplementary Data**

**S1 Table**. The values shown in Table S1 were used to generate Figure 3. This experiment was completed on three additional occasions with similar results.

| Time (Minutes) | Viable phage count (PFU mL^-1^ ) |
| --- | --- |
| 0 | 2.1 x 10^4^ |
| 10 | 2.0 x 10^4^ |
| 20 | 2.5 x 10^4^ |
| 30 | 1.8 x 10^5^ |
| 40 | 2.0 x 10^6^ |
| 50 | 3.9 x 10^6^ |
| 60 | 3.0 x 10^7^ |
| 70 | 1.8 x 10^8^ |
| 80 | 2.4 x 10^8^ |
| 90 | 2.8 x 10^8^ |
| 100 | 2.9 x 10^8^ |
| 110 | 3.0 x 10^8^ |
| 120 | 3.1 x 10^8^ |
| 150 | 3.0 x 10^8^ |
