# Supplementary material for: Characterization and formulation into solid dosage forms of a novel bacteriophage lytic against Klebsiella oxytoca
Source: PLoS One. 2017 Aug 17;12(8):e0183510. doi: 10.1371/journal.pone.0183510 (PMC5560551; doi:10.1371/journal.pone.0183510)
Supplement: S2 Table — The values shown in S2 Table were calculated and used to generate Fig 4. (DOCX) [file pone.0183510.s002.docx]

**Supplementary Data**

**S2 Table** Because the measurements for each of the triplicates in figure 4 were very similar, it was not possible to adequately represent the error bars for standard deviation. The values shown in Table S2 were calculated and used to generate Figure 4.

**Simulated Gastric Fluid**

| Time (minutes) | Plate 1  (PFU mL^-1^ ) | Plate 2  (PFU mL^-1^ ) | Plate 3  (PFU mL^-1^ ) | Average (PFU mL^-1^ ) | Standard deviation (PFU mL^-1^ ) |
| --- | --- | --- | --- | --- | --- |
| 0 | 8.5 x 10^6^ | 8.4 x 10^6^ | 8.45 x 10^6^ | 8.45 x 10^6^ | 5.0 x 10^4^ |
| 15 | 7.9 x 10^5^ | 8.0 x 10^5^ | 8.1 x 10^5^ | 8.0 x 10^5^ | 1.0 x 10^4^ |
| 30 | 4.2 x 10^5^ | 4.5 x 10^5^ | 4.3 x 10^5^ | 4.3 x 10^5^ | 1.5 x 10^4^ |
| 45 | 3.0 x 10^4^ | 2.8 x 10^4^ | 2.9 x 10^4^ | 2.9 x 10^4^ | 1.0 x 10^3^ |
| 60 | 1.5 x 10^4^ | 1.3 x 10^4^ | 1.45 x 10^4^ | 1.42 x 10^4^ | 1.0 x 10^3^ |
| 75 | 8.5 x 10^3^ | 8.7 x 10^3^ | 8.6 x 10^3^ | 8.6 x 10^3^ | 1.0 x 10^2^ |
| 90 | 3.9 x 10^3^ | 4.0 x 10^3^ | 3.9 x 10^3^ | 3.97 x 10^3^ | 5.7 x 10^1^ |

**Bile salts solution**

| Time (minutes) | Plate 1  (PFU mL^-1^ ) | Plate 2  (PFU mL^-1^ ) | Plate 3  (PFU mL^-1^ ) | Average (PFU mL^-1^ ) | Standard deviation (PFU mL^-1^ ) |
| --- | --- | --- | --- | --- | --- |
| 0 | 7.7 x 10^8^ | 7.6 x 10^8^ | 7.4 x 10^8^ | 7.57 x 10^8^ | 1.53 x 10^7^ |
| 15 | 7.4 x 10^8^ | 7.7 x 10^8^ | 7.8 x 10^8^ | 7.63 x 10^8^ | 2.08 x 10^7^ |
| 30 | 7.2 x 10^8^ | 7.3 x 10^8^ | 7.5 x 10^8^ | 7.33 x 10^8^ | 1.53 x 10^7^ |
| 45 | 7.2 x 10^8^ | 7.0 x 10^8^ | 7.3 x 10^8^ | 7.17 x 10^8^ | 1.53 x 10^7^ |
| 60 | 7.1 x 10^8^ | 6.9 x 10^8^ | 7.2 x 10^8^ | 7.07 x 10^8^ | 1.53 x 10^7^ |
| 75 | 6.8 x 10^8^ | 6.9 x 10^8^ | 6.9 x 10^8^ | 6.87 x 10^8^ | 5.77 x 10^6^ |
| 90 | 6.5 x 10^8^ | 6.6 x 10^8^ | 6.7 x 10^8^ | 6.60 x 10^8^ | 1.0 x 10^7^ |
