# Supplementary material for: Characterization and formulation into solid dosage forms of a novel bacteriophage lytic against Klebsiella oxytoca
Source: PLoS One. 2017 Aug 17;12(8):e0183510. doi: 10.1371/journal.pone.0183510 (PMC5560551; doi:10.1371/journal.pone.0183510)
Supplement: S3 Table — (DOCX) [file pone.0183510.s003.docx]

**Supplementary Data**

**S3 Table**. These values were used to generate Figure 7.

**Simulated Gastric Fluid**

| Time (Days) | Plate 1  (PFU mL^-1^ ) | Plate 2  (PFU mL^-1^ ) | Plate 3  (PFU mL^-1^ ) | Average (PFU mL^-1^ ) | Standard deviation (PFU mL^-1^ ) |
| --- | --- | --- | --- | --- | --- |
| 0 | 1.05 x 10^8^ | 1.06 x 10^8^ | 1.06 x 10^8^ | 1.06 x 10^8^ | 5.77 x 10^5^ |
| 7 | 9.80 x 10^7^ | 9.00 x 10^7^ | 9.60 x 10^7^ | 9.47 x 10^7^ | 4.16 x 10^6^ |
| 14 | 8.80 x 10^7^ | 8.7 x 10^7^ | 8.9 x 10^7^ | 8.8 x 10^7^ | 1.00 x 10^6^ |
| 21 | 8.0 x 10^7^ | 7.9 x 10^7^ | 7.81 x 10^7^ | 7.9 x 10^7^ | 9.5 x 10^5^ |
| 28 | 7.2 x 10^7^ | 7.3 x 10^7^ | 7.1 x 10^7^ | 7.2 x 10^7^ | 1.0 x 10^6^ |
| 35 | 6.9 x 10^7^ | 6.7 x 10^7^ | 6.8 x 10^7^ | 6.8 x 10^7^ | 1.0 x 10^6^ |
| 42 | 5.3 x 10^7^ | 5.6 x 10^7^ | 5.5 x 10^7^ | 5.47 x 10^7^ | 1.53 x 10^6^ |
| 49 | 4.2x 10^7^ | 3.9 x 10^7^ | 4.1 x 10^7^ | 4.07 x 10^7^ | 1.53 x 10^6^ |
